# Robust Hydrogen Production *via* Pickering Interfacial Catalytic Photoreforming of n-Octanol-Water Biphasic System

**DOI:** 10.3389/fchem.2021.712453

**Published:** 2021-07-22

**Authors:** Chao Wang, Weilin Zhong, Suqing Peng, Jingtao Zhang, Riyang Shu, Zhipeng Tian, Qingbin Song, Ying Chen

**Affiliations:** ^1^ Guangdong Provincial Key Laboratory on Functional Soft Condensed Matter, School of Materials and Energy, Guangdong University of Technology, Guangzhou, China; ^2^ Macau Environmental Research Institute, Macau University of Science and Technology, Macau, China

**Keywords:** hydrogen production, photoreforming, pickering interfacial catalysis, microfluidics, Pt/TiO_2_ nanosheets

## Abstract

Pickering emulsion offers a promising platform for conducting interfacial reactions between immiscible reagents; it is particularly suitable for hydrogen production by photoreforming of non-water soluble biomass liquid and water. Herein, Pt-promoted (001)-facet-dominated anatase TiO_2_ nanosheets were synthesized by a hydrothermal route associated with microfluidic technology for high activity and metal dispersion, and selective surface modification was carried out for preparing Janus particles. Photoreforming hydrogen production through n-octanol and water that formed O/W microemulsion with an average diameter of 540 µm was achieved to obtain amphiphilic catalyst. The as-prepared 2D Janus-type catalysts exhibited remarkably stable emulsification performance as well as photocatalytic activity. This finding indicates that triethoxyfluorosilane had negligible impact on the catalytic performance, yet provided a remarkable benefit to large specific surface area at microemulsion interface, thereby enhancing the H_2_ yield up to 2003 μmol/g. The cyclic experiments indicate that the decrease in cyclic performance was more likely to be caused by the coalescence of the microemulsion rather than the decrease in catalytic activity, and the microemulsion could be easily recovered by simply hand shaking to more than 96% of the initial performance.

## Introduction

Solar-driven hydrogen production from biomass, as a clean and renewable-based process, holds great promise for reducing the demand on fossil resources and meeting the growing energy demand (Goyal et al., 2008; Bhattacharya et al., 2017; Zhang et al., 2017). Compared with thermochemical methods, photocatalysis could be carried out in surrounding conditions under solar irradiation with less total energy input and reduced carbon footprint (Bridgwater, 2003; Schneider et al., 2014; Liao et al., 2019). Especially, photoreforming combines water reduction and bioderived organic compound oxidation into a single process with enhanced reaction kinetics and efficiency toward hydrogen production. Although many biomass liquids could produce hydrogen via photoreforming, a large number of bioderived reagents immiscible with water (such as long chain alcohols, LCAs) cannot easily and efficiently produce hydrogen in the formation of biphasic liquid systems (Murdoch et al., 2011; Fessi et al., 2019). The formed biphasic system with low mutual solubility and stratification between reagents often displays low catalytic activity due to the poor mass/heat transfer of the reagents to the catalyst surface.

**GRAPHICAL ABSTRACT:**
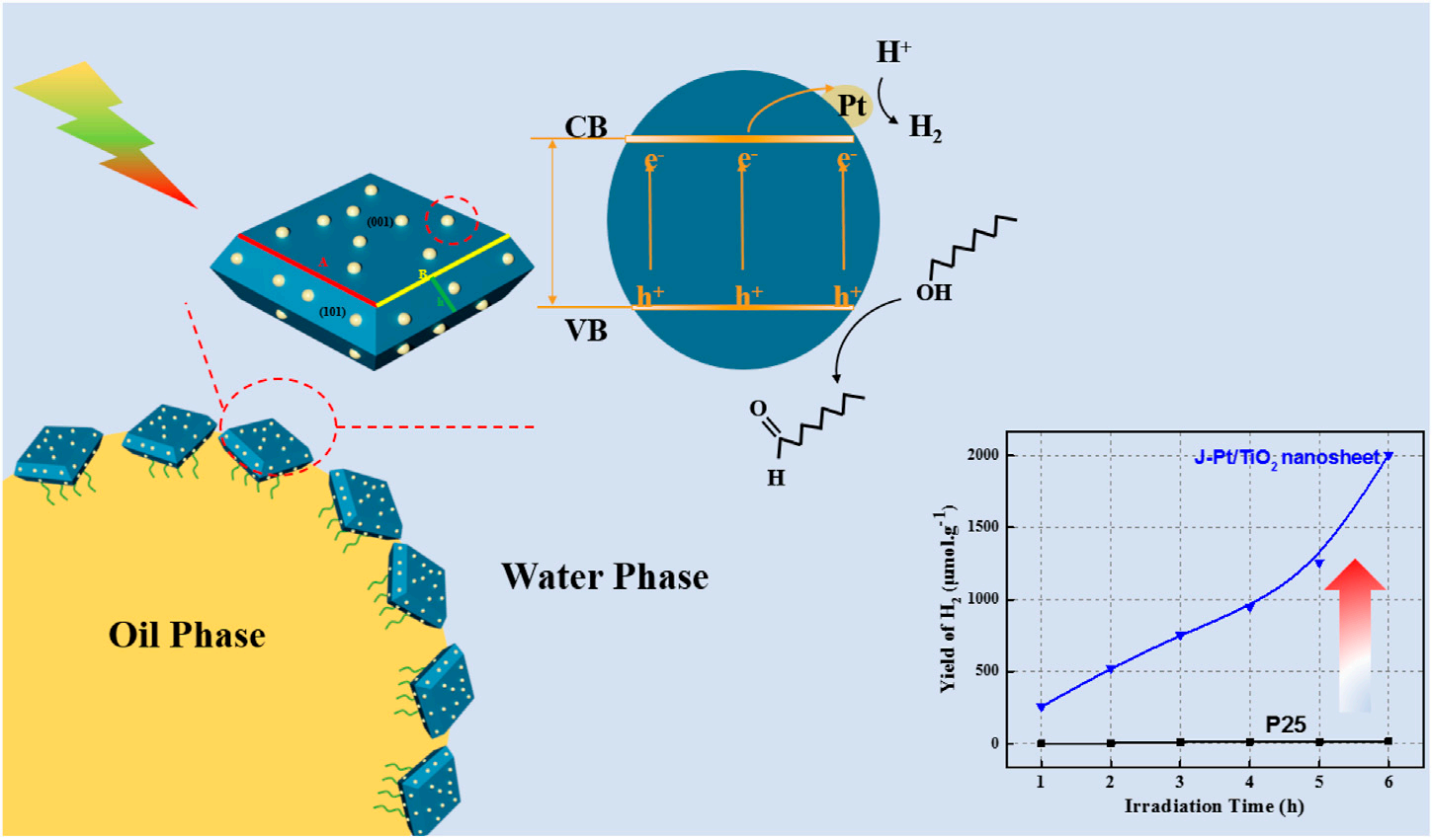


A conventional idea for using surfactants or surface-active polymers is discouraged in this case because their separation after reaction might result in serious burdens (Mathiesen et al., 2011). As an alternative, Pickering interfacial catalysis (PIC) has emerged as a powerful approach for implementing multiphasic reactions between immiscible reagents via heterogeneous catalysts (Pera-Titus et al., 2015; Yang et al., 2017). The concept of PIC encompasses the fabrication of amphiphilic nanoparticles (NPs) or the so called “Janus-type” particle with catalytic active centers acting as emulsifier and catalyst simultaneously. The constructed Pickering emulsions, in which mesoscopic domains of multiple liquid phases are stabilized by amphipathic solid particles at the liquid/liquid (L/L) interface with neither solvents nor surfactants, enable the effective diffusion of the liquid molecules with extremely large ratio of liquid–fluid interface to volume (Ding et al., 2018). Such features are considered to significantly enhance chemical reaction efficiency between the multiple liquid phases (Li et al., 2019). Except for photocatalytic activity, interfacial catalysis also relies on the formation of a dense thin film formed by self-assembled NPs at the L/L interface; it prevents the droplets from coalescence (Kaewsaneha et al., 2013). In our previous studies, spherical anatase TiO_2_-based photocatalysts were confirmed to display an excellent PIC photoreforming hydrogen production performance (Bu et al., 2019; Wang et al., 2019). Single-crystalline anatase TiO_2_ nanosheets with high percentage of reactive (001) facets might further enhance photocatalytic activity given their high surface energy toward efficient dissociative adsorption of reactant molecules (Selloni, 2008). 2D morphology could enhance the emulsion stability by multilayer tiling that increases interfacial coverage (Gonzalez Ortiz et al., 2020). In addition, noble metal loading, such as Pt, Ru, and Pd, could form a Schottky junction between the metal (especially for Pt and Pd) and semiconductor, thereby promoting the transfer of photogenerated electrons from n-type semiconductor to metal as a unidirectional charge pump to reduce the recombination of the electrons and holes (Bahruji et al., 2011; Bai et al., 2015; Gu et al., 2016; Cheng et al., 2019; Fu and Ren, 2020). It is essential to understand that high dispersion of noble metal could significantly improve accessibility of active sites. Photo-deposition sparked interest in catalyst fabrication due to its ability to achieve high dispersion of metal loading since it was proposed by Kraeutler and Bard in 1978 (Wenderich and Mul, 2016). Compared with chemical reduction and electro-deposition, photo-deposition is a simple operation, only requiring light illumination upon a slurry reactor; this method can regulate the distribution, size, and oxidation state of NPs by careful selection of preparation conditions (Busser et al., 2012). In recent years, microfluidic technology has been widely used to synthesize catalyst NPs given its small volume, high operation speed, and small length scale for accurate control of the synthesis parameters (van den Berg et al., 2010). Especially for photo-deposition, microfluidic technology can perfectly realize the advantages of this method through precise flow control in confined space.

We presented novel Janus-type Pt/TiO_2_ nanosheets with highly dominated (001) facets, where Pt metal particles were ultrafast photo-deposited on the TiO_2_ nanosheets in a transparent polydimethylsiloxane (PDMS) microfluidic device and selective surface modification was implemented to obtain Janus particles. Pickering emulsion can be obtained by self-assembly of Janus particles to the n-octanol/water interface. This photoreforming system with increased reaction surface area demonstrated excellent hydrogen production performance. Finally, the cyclic performance of the Janus Pt/TiO_2_ nanosheets was evaluated to examine their stability.

## Experimental

### Chemicals and Reagents

All reagents used were of analytical grade and used without further purification (Aladdin Industrial Corporation, Shanghai, China). In all experiments, the deionized water was obtained from Millipore Milli-Q ultrapure water purification system with a resistivity larger than 18.2 MΩ.

### Preparation of Catalyst

A high (001)-facet-dominated anatase TiO_2_ nanosheet was synthesized by a typical hydrothermal route. A total of 4 ml of 40% hydrofluoric acid (HF) solution was added dropwise carefully to 25 ml of tetra-butyl ortho-titanate (Ti(OC_4_H_9_)_4_, TBOT). Then, the mixture was transferred into a 100 ml Teflon-lined autoclave for a hydrothermal treatment at 200°C for 24 h. Subsequently, the sample was centrifuged, washed with deionized water and ethanol alternately to remove impurities, and then dried at 60°C for 24 h. The obtained (001) TiO_2_ sample was denoted as TiO_2_ nanosheet (TNS). P25 was purchased from Shanghai Macklin Biochemical Co., Ltd. as a comparison.

**FIGURE 1:**
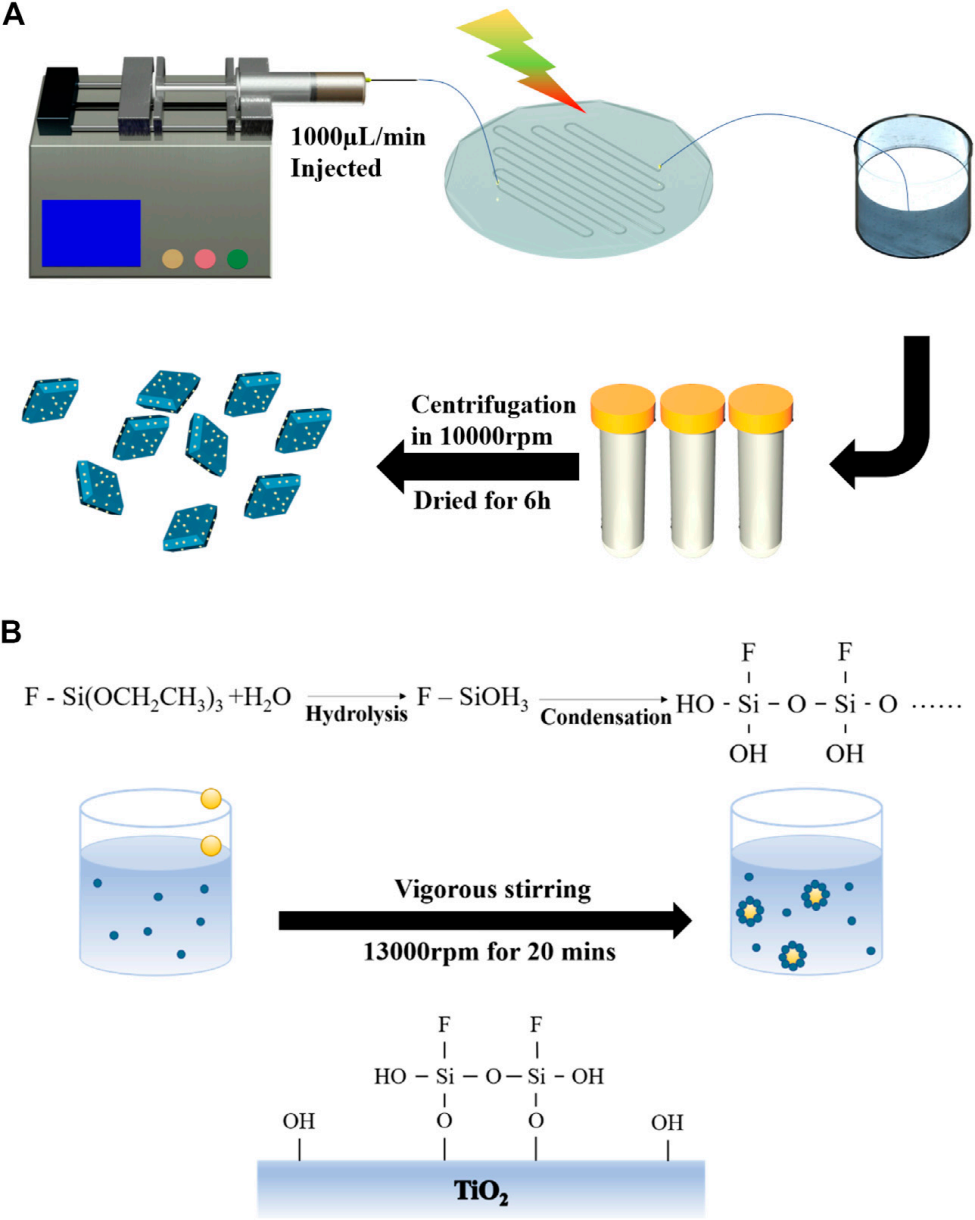
Fabrication of Janus-type Pt/TiO_2_ nanosheets: **(A)** ultrafast and highly dispersed loading of Pt on TiO_2_ nanosheets using microfluidic technology; **(B)** selective surface modification of Pt/TNS.

Generally, the photo-generated electrons were transferred to the surface of TNS during the ultraviolet illumination, where PtCl_6_^2-^ ions were reduced to metallic Pt (Lakshminarasimhan et al., 2012). In the confined space inside the microfluidic channel, the precursors were evenly and fully mixed, resulting in even and efficient loading (Figure 1A). Therefore, Pt metal NPs using microfluidic synthesis with better control of the time and spatial distribution result in better size homogeneity than typical reactors. Photochemical reduction deposition of Pt was carried out in a PDMS microfluidic device(Wang et al., 2018). The hydraulic diameter of the microchannel in the fabricated microfluidic chip was 850 μm, and the total length of the channel was 550 mm. An ultraviolet irradiation (30 W, 365 nm) was used to illuminate the transparent chip. In this experiment, 0.1 g TNS powder was added into 50 ml of chloroplatinic acid aqueous solution (with a concentration ca. 0.02 wt%) and transferred into a 50 ml injector after ultrasonic dispersion. The suspension was injected into the microchannel at a feeding rate of 450 μl/min through an injection pump (TYD01, Lead Fluid Technology Co., Ltd.). Prior to irradiation, the reactant mixture was degassed by bubbling nitrogen gas for 30 min. The suspension after reaction was collected in a beaker; then, the sample was subjected to centrifuge, washed, and finally dried at 60°C for 24 h. The obtained final sample prepared by microfluidic technology was denoted as Pt/TNS. Another sample was prepared by a conventional continuous stirring and irradiation with the same conditions to further confirm the advantage of the microfluidic; it was denoted as Pt/TNS-S.

### Fabrication of Janus-Type Pt/TNS

Partial hydrophobic modification by a Pickering emulsion method was carried out to achieve surface anisotropic properties of Pt/TNS (Liu et al., 2008). An amount of 0.1 gas-prepared Pt/TNS powder was dispersed in 60 ml deionized water. After ultrasonic treatment and stirring, 10 ml toluene solution containing 0.12 mol/L triethoxyfluorosilane (TEFS, C_6_H_15_FO_3_Si) was added dropwise to the suspension under stirring at 13,000 rpm with a high-performance disperser for 20 min (IKA T18, Ultra Turrax, Co., Ltd. Germany). Subsequently, the mixture was aged for 24 h. Then, the modified particles were separated from the solvent by centrifugation, followed by washing with ethanol and deionized water alternately to remove the residual reagents. The obtained sample was dried at 60°C for 24 h and denoted as J-Pt/TNS.

### Catalyst Characterizations

The crystal phase and microstructure of the catalyst were determined by X-ray powder diffraction (XRD) using Shimadzu XRD-6000 powder diffractometer (at 40 kV and 30 mA with Cu Kα-ray, with λ = 0.1541 nm). The detailed morphologies were observed by a high-resolution transmission electron microscope (HRTEM, Talos F200S). Fourier transform infrared (FTIR) spectra were acquired in air at room temperature on an FTIR spectrometer (Thermo Scientific Nicolet 6,700). Raman measurement was carried out using a Raman spectroscopy (NOST FEX, Korea) with 532 nm laser excitation. The facial element status and chemical composition of particles were detected by X-ray photoelectron spectroscopy (XPS, Escalab 250Xi, Thermo Fisher) analysis. UV-Vis diffused reflectance spectra of the samples were recorded by a UV-Vis spectrophotometer (UV-3600 plus, Shimadzu, Japan), where BaSO_4_ was applied as a reflectance standard. An optical microscopy equipped with a digital camera was installed under an inverted optical microscope (BDS400, Optex Co. Ltd. China) for observing Pickering emulsion. Furthermore, the stability of the emulsion droplet was confirmed by dispersion stability analyzer (TURBISCAN LAB). Thermal Gravimetric Analysis (TGA, TA TGA5500) was applied to estimate the amount of the grafted reagent.

### Photocatalytic Hydrogen Production

The photocatalytic hydrogen production performance was evaluated by a photocatalytic system with a light source (320–780 nm, Philae technology Co. Ltd. Beijing, China) vertically arranged 10 cm directly above the glass reactor. The focused light intensity and areas were ca. 350 mW/cm^2^ and 0.28 cm^2^, respectively. A 0.2 g catalyst sample was suspended in the mixture of 80 ml deionized water and 20 ml n-octanol in a vacuumed duplex Pyrex flask (−0.1 MPa, ambient temperature) under ultrasonic to generate emulsion. A hydrogen production system was directly connected to an online gas chromatographer (GC-2014c AT, Shimadzu, Japan, TCD, and nitrogen as a carrier gas and 5 Å molecular sieve column). The AQY at 350 nm was calculated according to the equations (Yuan et al., 2016):

$$AQY\left(\%\right)=\frac{number\;of\;reacted\;electrons}{number\;of\;incident\;photos}\times 100$$ (1)

## Results and Discussions

### Characterizations of Synthesized Samples

**FIGURE 2:**
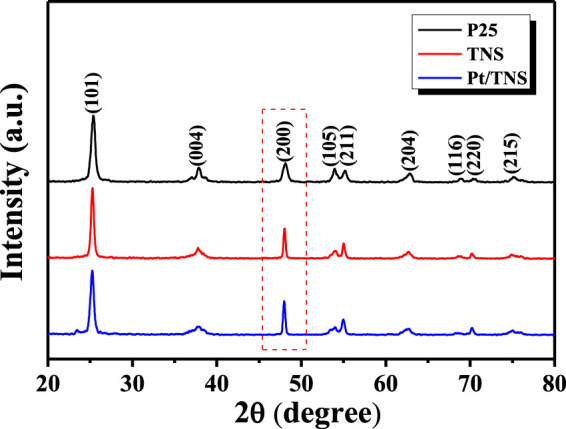
XRD patterns of anatase P25, TNS, and Pt/TNS.

Anatase TiO_2_ could exhibit higher photocatalytic activities toward hydrogen production than rutile and brookite(Zhang et al., 2008). In Figure 2, the diffraction peaks of all samples appeared (JCPDS No. 21–1,272) at the 2θ diffraction angles of 25.3°, 37.8°, 48.0°, 53.9°, 55.0°, and 62.6° that could be attributed to the (101), (004), (200), (105), (211), and (204) anatase lattice planes, respectively. The peak intensity of (200) Bragg peak significantly increased, and its full width at half-maximum became narrow, revealing that the side length of the sample in (001) direction was increased by adding HF. The thickness of TNS in the (001) direction decreased because the intensity of (004) diffraction peaks was reduced and the full width at half-maximum was broadened in (001) direction(Tian et al., 2012). Both changes in (004) and (200) peaks were due to the high percentage of exposed (001) facets caused by the HF treatment. No evident diffraction peaks of Pt were observed mainly due to the low load and high dispersion(Yu et al., 2010). Raman spectrum illumination by 532 nm was also carried out to examine the bond structure of the samples (Supplementary Figure 2, Supporting Information).

**FIGURE 3:**
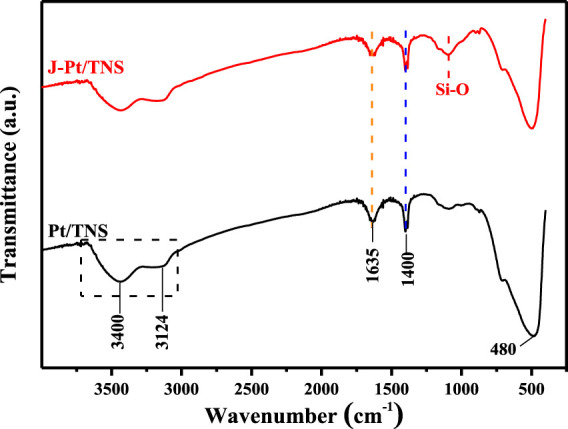
FTIR spectra of the J-Pt/TNS and Pt/TNS.

Figure 3 shows that the peaks located at 3,400 and 3,124 cm^−1^ were mainly attributed to the stretching vibrations of hydroxyl groups of Ti-OH interacting with physisorbed water molecules through hydrogen bonding (Bezrodna et al., 2003; Li et al., 2005). The sharp peak in 1,635 cm^−1^ was characterized as the physically absorbed H-O-H in water, and another sharp peak located at 1,400 cm^−1^ accounted for the absorption of CO_2_ in the atmosphere to form CO_3_^2-^ or HCO_3_^2-^ ions (Li et al., 2004). The increased peak width at the wavenumber near 1,100 cm^−1^ of J-Pt/TNS might be caused by the hydrolysis procedure of TEFS, creating a large number of Si-O functional groups on J-Pt/TNS. This finding convincingly demonstrated that the functional groups of silane coupling agents were successfully grafted on the surface of Pt/TNS (Jung and Park, 2000). The strong intensity peak near 480 cm^−1^ corresponded to Ti-O-Ti in titanium dioxide (Oliveira et al., 2008). The amount of grafted TEFS on J-Pt/TNS could be determined by thermal gravimetric analysis (Supplementary Figure 3, Supporting Information).

**FIGURE 4:**
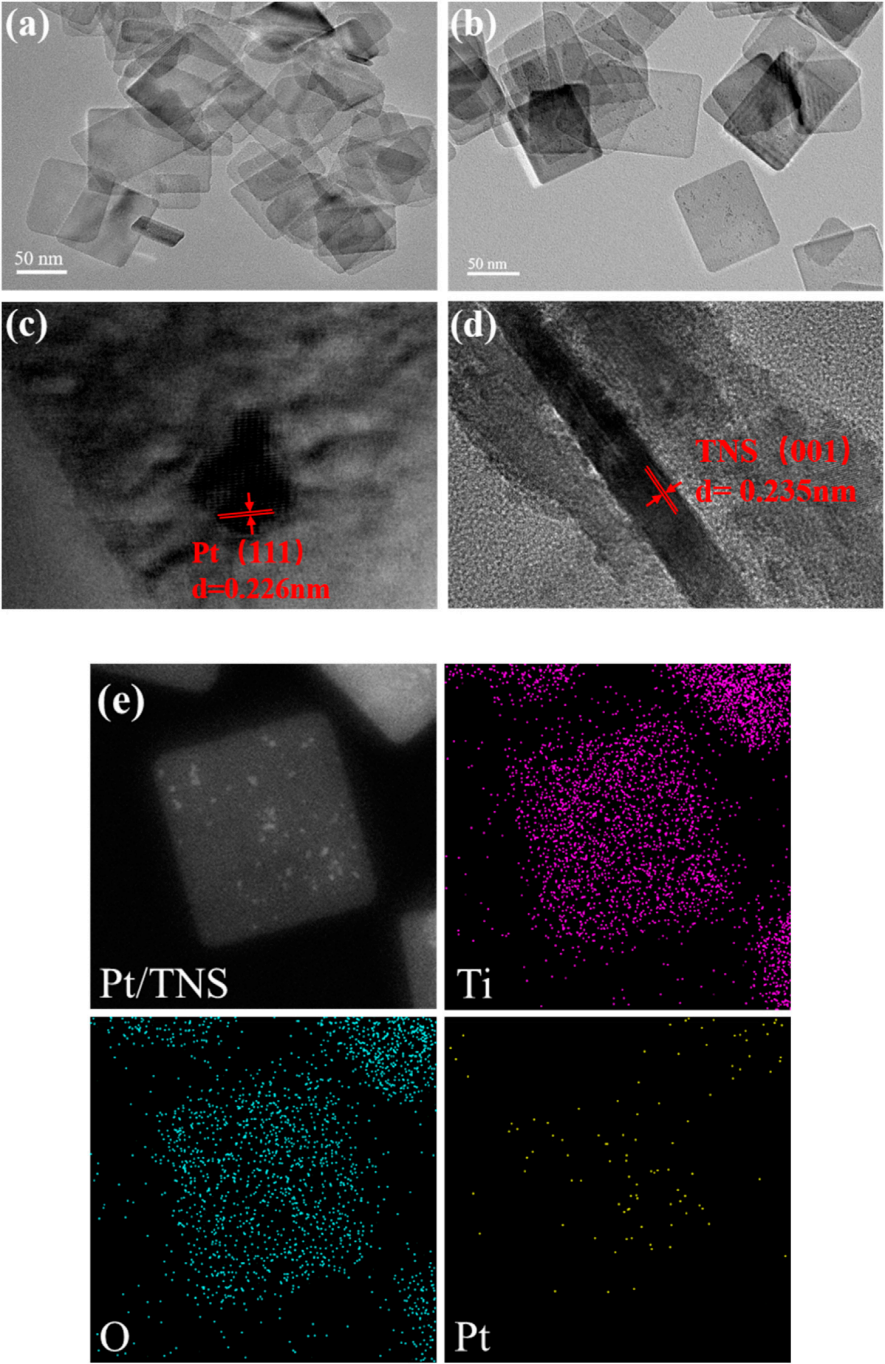
HRTEM images of **(A)** TNS, **(B)** Pt/TNS, **(C)** lattice fringes Pt, **(D)** lattice fringes TNS, and **(E)** element distributions of Pt/TNS.

In Figure 4A, the as-prepared TiO_2_ nanosheet exhibited a clear sheet-like structure with an average side length of ca. 70–90 nm and thickness of ca. 8 nm. Pt nanoparticles were evenly distributed on the TNS with the average diameter of ca. 2–3 nm (Figure 4B) by using the microfluidic photo-reduction technology, and the distribution of the Pt particles on Pt/TNS is shown in Figure S4. The lattice fringes of Pt nanoparticles and TiO_2_ nanosheets (Figures 4C,D) were 2.26 and 2.35 Å, corresponding to 111) planes of Pt and (001) planes of TNS, respectively(Xiang et al., 2010; Yu et al., 2010; Yu et al., 2017). Therefore, the percentage of the exposed (001) facets of TNS was approximately 81.9%(Yuan et al., 2016). Element mapping images shown in Figure 4E further suggested the successful and uniform loading of Pt on TNS. Moreover, the distribution of Ti, O, and Pt elements on the surface of the Pt/TNS and the molar ratio of Pt to Ti on sample was ca. 4.75%, which was obtained by the microfluidic preparation method, and those in the Pt/TNS-S was ca. 2.01%, shown in Supplementary Figure 1.

**FIGURE 5:**
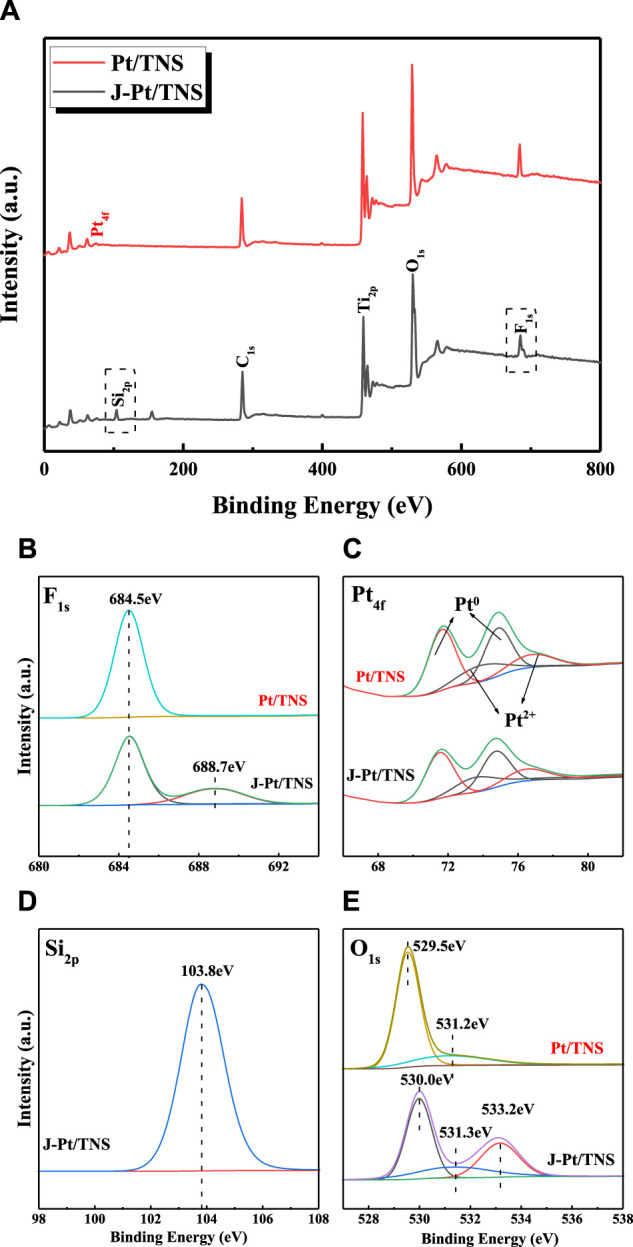
Graphic diagram of the **(A)** survey and high-resolution XPS spectra of **(B)** F 1s, **(C)** Pt 4f, **(D)** Si 2p for J-Pt/TNS, and **(E)** O 1s spectra of Pt/TNS and J–Pt/TNS.

Surface chemical elements and electron energy state by X-ray photoelectron spectroscopy (XPS) analysis are shown in Figures 5A–E. All peak positions were calibrated with C 1s at 284.8 eV prior to fitting. Pt, C, Ti, O, and F were detected as the main compositions for each case. Ti 2p_3/2_ and Ti 2p_1/2_ in Pt/TNS could be confirmed at binding energies of 458.7 and 464.5 eV, respectively. In Figure 5B, element F could only be detected at binding energy of 684.5 eV, indicating that F might exist in the form of fluoride ion from surface fluoride (Ti–F) generated from ligand exchange reaction between F^−^ and the surface hydroxyl on TNS surface (Yu et al., 2002; Murcia et al., 2015). The peak attributed to covalent F atom in Si-F bond centered around 688.7 eV further confirmed the successful grafting of TEFS onto the photocatalyst (Kuwahara et al., 2009). Si_2p_ at 103.8 eV corresponded to O-Si-O in J-Pt/TNS, indicating the presence of organo-silanol generated by hydrolysis of silane coupling agent combined with surface hydroxyl group of Pt/TNS, and the procedure of the formation of O-Si-O is shown in Figure 1B (Acres et al., 2012). Figure 5E shows that O_1s_ spectra could be resolved into three peaks at 530.0, 531.3, and 533.2 eV for J-Pt/TNS. O_1s_ had peak shift in the first two degrees caused by the chemical environmental change, such as external reagent added resulting in the redistribution of electrons from the surface hydroxy. Particular O_1s_ characteristic peak at 533.2 eV for J-Pt/TNS was ascribed to the formed Si-O by hydrolysis of TEFS (Zhou et al., 2014; Bu et al., 2019). Anisotropic surface modification of Pt/TNS could bring about extra functional group, thereby distinguishing the spectra of F_1s_, O_1s_, and Si_2p_. In Figure 5C, four peaks were separated via Lorentzian–Gaussian fitting method. Pt^0^ was identified as Pt 4f_7/2_ at 71.7 and 74.9 eV for Pt/TNS and Pt 4f_5/2_ at 71.5 and 74.7 eV of J-Pt/TNS, which were synthesized by reduction of PtCl_6_^2-^ by photogenerated electrons. The identified peaks at 73.76 and 76.9 eV could be assigned to Pt^2+^, which was oxidized from Pt^0^ exposed in the atmosphere. Pt metallic state was predominant in the Pt/TNS(Young et al., 2008; Haselmann and Eder, 2017).

**FIGURE 6:**
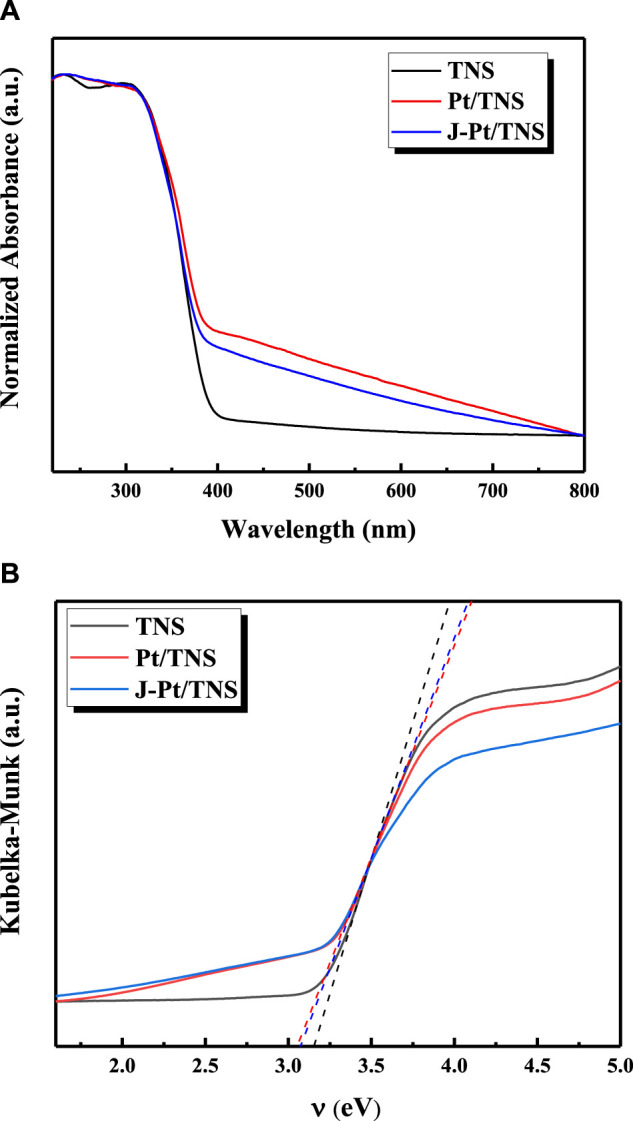
**(A)** UV-vis spectrum and **(B)** Tauc plot of the as-prepared samples.

In Figure 6A, significant absorption intensities at wavelengths shorter than 400 nm were observed for all samples; they could be assigned to the excitation of O_2p_ electron to Ti_3d_ level in anatase TiO_2_(Kumar et al., 2016). The absorption spectra of Pt/TNS and J-Pt/TNS samples appeared, thereby enhancing absorptions in the visible-light region because of the localized surface plasmon resonance (LSPR) from the surface Pt cluster; LSPR depended on the size, shape, and dielectric environment of the metal NPs (Langhammer et al., 2006; Lakshminarasimhan et al., 2012). The band gaps of each photocatalyst were calculated from the Tauc plot by mathematic methodology in Eq. 2, Eq. 3 from the UV-vis absorption spectra.

**FIGURE 7:**
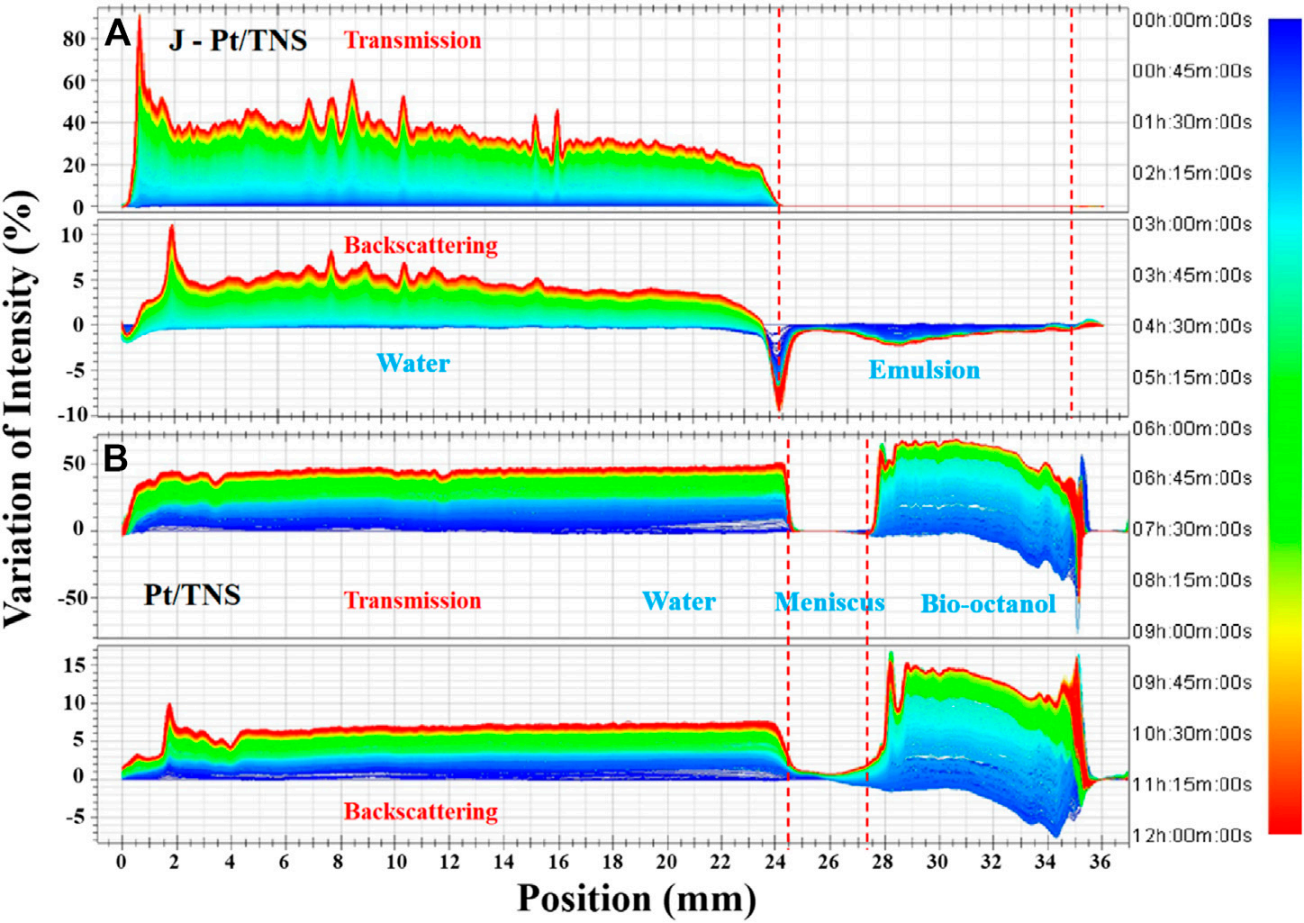
Variations of transmission and backscattering at 25°C for the system of **(A)** J-Pt/TNS and **(B)** Pt/TNS (stated bottom of bottle was 2 mm).

A=−lg(R) (2)

$$F\left(R\right)=\frac{{\left(1-R\right)}^{2}}{2R}$$ (3)

where *A* refers to the absorbance, and *R* is the reflectance (Lee et al., 2018) In Figure 7B, the band gap values of TNS, Pt/TNS, and J-Pt/TNS were 3.15, 3.05, and 3.07 eV, respectively, implying that Pt-loading reduced 3.5% band gap value of TiO_2_, and the surface modification hardly affected the band gap value of the photocatalysts.(Wang et al., 2019)

### Emulsification Property of J-Pt/TNS

**FIGURE 8:**
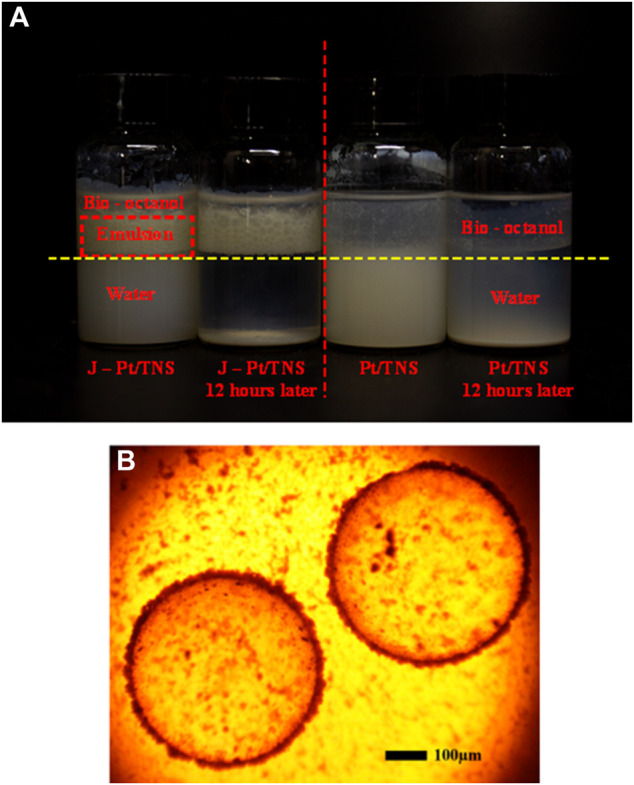
Photographs of **(A)** standings of emulsification and **(B)** microscopic image of emulsion droplets.

The stability of the microemulsion was one of the key factors that determine the reaction efficiency. Therefore, examining the emulsification properties of the catalyst is essential. A 10 ml of water and 5 ml of n-octanol were mixed with 0.3 wt% photocatalyst added in the bottle, and each mixture was sonicated for 5 min and shaken vigorously by hand to form O/W Pickering emulsion. A photograph of the mixture after emulsification and standing for 12 h is shown in Figure 8A, where the red dash line distinguished Pt/TNS and J-Pt/TNS, and the yellow dash line was the boundary of water and n-octanol. Evidently, the dispersion properties of Janus-type photocatalyst and Pt/TNS were different. At first, most Pt/TNS particles were suspended in the lower water phase instead of the upper n-octanol phase, and the situation of J-Pt/TNS was the opposite. Subsequently, the emulsion layer appeared between the water and n-octanol layer, and the droplets could be observed by optical microscope, as shown in Figure 8B. The average diameter was approximately 540 µm of the formed emulsion droplets. After 12 h standing, the emulsion constructed by J-Pt/TNS had an excellent stability. In addition, Pt/TNS mainly dispersed in the water phase and gradually sank to the bottom owing to agglomerations.

Monitoring the real-time destabilization of the formed emulsion is interesting for interfacial catalytic hydrogen production. However, bottle test or microscopy is unsuitable for detecting real-time coalescence of the droplets. Turbiscan analysis, as a nondestructive measure, could provide visualized real-time information on the gradual destabilization process of emulsion. Figure 7 shows that the transmission and backscattering profiles at time 0 were set as the baselines, and light intensities at various sample heights were recorded at set time intervals. The high transmission values at the bottom and the top positions were caused by the meniscus shape of solid–liquid and air–liquid interfaces. The change in Pt/TNS sample at various heights in the lower water layer was relatively gentle mainly because of less steric hindrance than J-Pt/TNS with graft groups on the surface. According to Figure 8A, the formed Pickering emulsion was mainly distributed in the upper oil phase, and slight changes in transmission intensities were observed in the upper oil phase for J-Pt/TNS. Moreover, the delta backscattering signals slightly decreased as the demulsification progressed. After analyzing the small changes in backscattering profiles in the upper layer according to the study of Mengual et al. (1999), the demulsification was induced by droplet coalescence and sedimentation. As a progressive decrease in backscattering in the initial period, coalescence was dominated in the emulsion. Then, the droplets migrated from the top to the bottom of the emulsion layer after coalescing droplets increased. This finding could be evidenced by the decrease in BS at the top and bottom. Finally, the demulsified droplets formed the water phase at the bottom (sedimentation), and the oil phase migrated toward the upper layer (clarification). Basically, the emulsion was stable within 12 h of testing.

### Photocatalytic H_2_ Production

**FIGURE 9:**
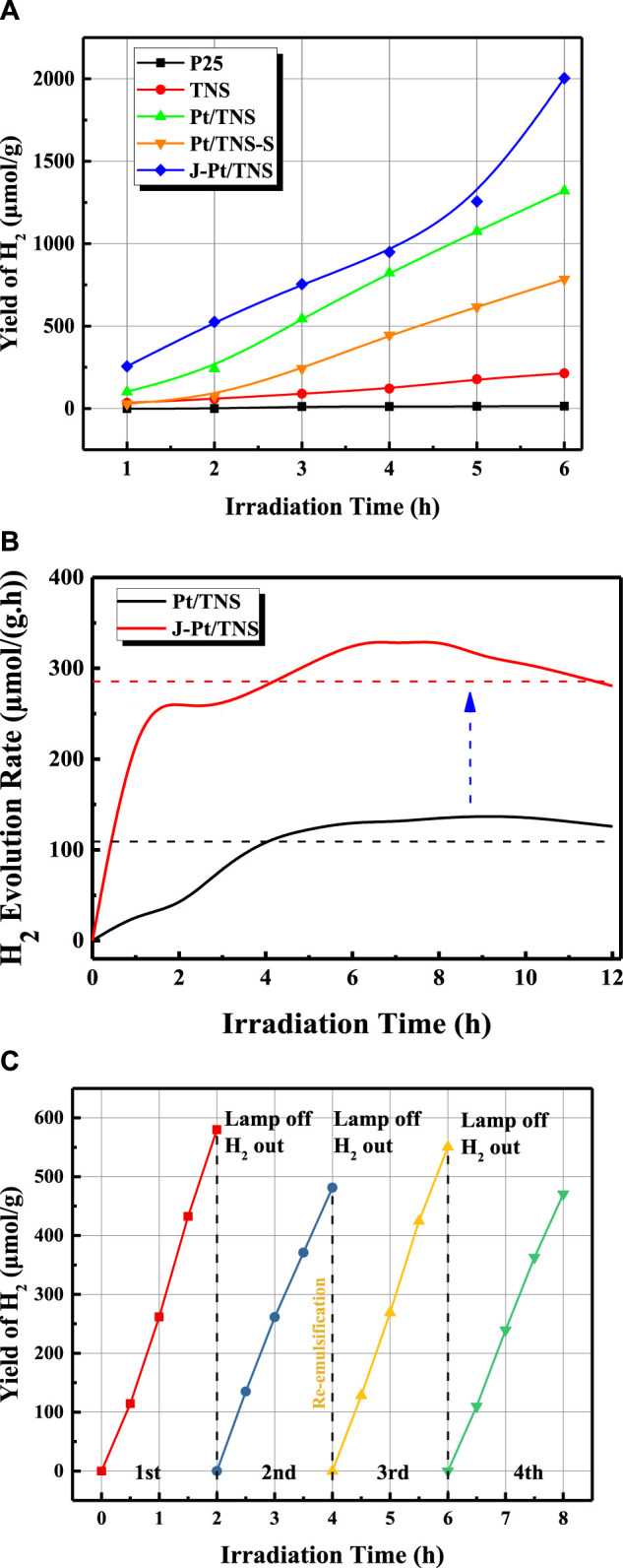
Diagram of **(A)** yield of H_2_ of P25, TNS, Pt/TNS-S, Pt/TNS, and J-Pt/TNS in 6 h, **(B)** hydrogen evolution rate of Pt/TNS and J-Pt/TNS in 12 h, and **(C)** cyclic performance of Pickering interfacial catalytic hydrogen production over J-Pt/TNS.

H_2_ production for 6 h is shown in Figure 9A. As expected, the produced hydrogen was increased with increasing irradiation time for all catalysts. Compared with commercial P25, the highly active crystal face of TNS surface was dominated by wet chemical etching, and theoretical studies in previous works indicated that the (001) surface with higher surface energy (0.98 J/m^2^) than (101) surface (0.49 J/m^2^) may be the dominant source of active sites for photocatalytic H_2_ production(Gong and Selloni, 2005; Yang et al., 2009; Yu et al., 2017). Therefore, the H_2_ production of TNS in the n-octanol and water system was nearly 14 times larger than that of P25, which was only 15 μmol/g after 6 h of irradiation. Our previous study discovered that the addition of a nonwater soluble biomass liquid to form a biphasic system could evidently improve H_2_ production performance compared with pure water splitting through photoreforming mechanism(Kudo and Miseki, 2009). Pt/TNS-S showed a lower yield of H_2_ than the hydrogen production of approximately 1,300 μmol/g of Pt/TNS due to its low molar ratio of Pt to Ti and poor dispersion of Pt nanoparticles according to the energy dispersive X-ray spectroscopy and HRTEM results, which meant lower active sites for hydrogen production Utilizing a Janus-type catalyst, H_2_ yield was promoted to 2003 μmol/g, which was 136.66, 36.42, 1.54, and 2.55 times that of P25, J-TiO_2_ (used in the previous study) (Wang et al., 2019), Pt/TNS, and Pt/TNS-S, respectively. Compared to pure water, the introduced of bio-octanol could effectively improve the yield of hydrogen, confirmed that the photogenerated electrons captured process by the surface reactants were not affected by the emulsion system. The AQY at 350 nm was calculated as 1.11% in J-Pt/TNS and 0.72% in Pt/TNS via Eq. 1. In fact, the shape of the catalyst particles governs the emulsifying performance at the interface (Gonzalez Ortiz et al., 2020). Compared with the spherical TiO_2_ acting as Pickering emulsifier, nonspherical particles could induce interface-mediated capillary forces with specific aspect ratios (Loudet et al., 2006). Furthermore, the continuous H_2_ production performance of Pt/TNS and J-Pt/TNS within 12 h is shown in Figure 9B, where the dashed line represented the average rate of 109 and 285 µmol/(g∙ h), respectively. The curve of Pt/TNS initially increased above the average value, and then remained stable after 4 h. In addition, J-Pt/TNS displayed a rapid increase in the first period, indicating the excellent performance of the initially “microreactor.” The H_2_ production rate of Pt/TNS and J-Pt/TNS started to decrease at approximately 10 and 7 h, respectively. The catalytic properties indicated that the decrease in Pt/TNS was more likely caused by the particle sedimentation rather than catalytic deactivation. In the case of J-Pt/TNS, emulsion stability played a more important role than photocatalytic activity (Bu et al., 2019). According to the result of the Turbiscan analysis, coalescence of the droplet was still the dominant phenomenon in the upper emulsion phase within the reaction. After the coalescence occurred, the emulsion droplet increased until they burst, followed by aggregation and sedimentation of the J-Pt/TNS particles from the emulsion phase to the water phase, resulting in the reduction in the reaction area at the emulsion interface. It was this Janus-type modification method that increased the contact surface area between the photocatalyst and the oil-water interface, since it could open the source of transport channels for the molecules involved in the process redox reactions and effectively separate the photogenerated charge carriers, thus reducing the electron-hole pairs recombination (Khan et al., 2019). Besides, with the high dispersion of Pt nanoparticle provided more active site compared to agglomerated states (Sui et al., 2017). Both of above factor was accounted for the improvement of hydrogen production in biphasic system.The cyclic performance of the J-Pt/TNS was evaluated with each cyclic irradiation for 2 h without stirring. In each regeneration process, the lamp was turned off for 30 min, and the vacuum pump was turned on to extract H_2_ for the substrates to re-oxidize the photocatalyst. In Figure 9C, the initial H_2_ yield was decreased from 579.95 μmol/g to 481.50 μmol/g with a reduction of 16.9% in the first and second cycle mainly due to the reduction in the catalytic specific surface area at the emulsion interface. Surprisingly, after reemulsification by simple hand shaking between the second and third cycle, the initial amount of H_2_ production was recovered to 550.64 and 470.95 μmol/g in the third and fourth cycles, respectively. Compared with the initial value in the first cycle, the initial hydrogen yield in the third cycle was only reduced by 3.8% after hand shaking for reemulsification. The slight difference in the initial H_2_ production amount between the first and the third cycle revealed the efficiency of reemulsification based on the understanding of the intrinsic properties toward photocatalytic hydrogen production.

## Conclusion

In summary, a novel (001)-facet-dominated Janus-type Pt/TNS was synthesized by a hydrothermal route combined with a photo-reduction route by using microfluidic technology. Through various characteristic approaches, Pt particles were found to be highly distributed on active (001)-facet-dominated anatase TiO_2_ nanosheets. Under the optical microscope and stability examination, the amphiphilic J-Pt/TNS particles displayed a stable emulsification performance at the n-octanol/water interface. Even if the surface was partially covered by the TEFS groups, the photocatalytic activity of J was still maintained at a high level because the maximum hydrogen production yield reached 2003 μmol/g in 6 h, which was nearly 140 times that of the commercial P25. In addition, the PIC system could be easily recovered to more than 96% of the initial performance only by hand shaking for re-emulsification. An efficient approach for using Janus-photocatalyst with highly dispersed metals in this work might pave a new path to enhance hydrogen production from biphasic photoreforming of non-water soluble liquids.

### Supporting Information

The diagram of (a) the molar ratio of the photo-reduced Pt particles on the TNS with variable feeding rate and (b-c) HRTEM of Pt/TNS-S and Pt/TNS, respectively (Supplementary Figure 1); Raman spectra of TNS, Pt/TNS, and J–Pt/TNS stimulated by 532 nm laser (Supplementary Figure 2); TGA and DTG curves of Pt/TNS and J-Pt/TNS (Supplementary Figure 3).

Mean diameter of Pt particles of Pt/TNS (Supplementary Figure 4).

## Data Availability

The original contributions presented in the study are included in the article/Supplementary Material, further inquiries can be directed to the corresponding author.
