# Supplementary material for: Robust Hydrogen Production via Pickering Interfacial Catalytic Photoreforming of n-Octanol-Water Biphasic System
Source: Front Chem. 2021 Jul 22;9:712453. doi: 10.3389/fchem.2021.712453 (PMC8339705; doi:10.3389/fchem.2021.712453)
Supplement: Supplementary file 1 [file DataSheet1.docx]

**Supporting Information**

**Robust Hydrogen Production via Pickering interfacial Catalytic Photoreforming of n-Octanol-Water Dual-Phasic System**

Chao Wang^1^, Weilin Zhong^1^, Suqing Peng^1^, Jingtao Zhang^1^, Riyang Shu^1^, Zhipeng Tian^1^, Qingbin Song^2^, Ying Chen^1,*^

1. Guangdong Provincial Key Laboratory on Functional Soft Condensed Matter, School of Materials and Energy, Guangdong University of Technology, Guangzhou 510006, China.

2. Macau Environmental Research Institute, Macau University of Science and Technology, Macau SAR, China.

***Corresponding Author**: Ying Chen

**E-mail**: chenying@gdut.edu.cn

**Tel**: +86-20-39322570

Number of pages: 7

Number of figures: 4

**S1. Design of Microfluidics Device**

Photo-deposition by reductive metal ions on the semiconductor is known as a very fast chemical process, and parameters can be controlled more precisely applying micromixers via microchannels.^1^ Generally, Reynolds number (Re) that gives a measure of inertial forces to viscous forces ratio depicting flow conditions (shown in eq. S1). At the low fluid velocity, mixing of precursors in a straight microchannel is inefficient, which would reduce the amount of the Pt-loaded. Thus, we designed some Dean-flow patterns to improve the micro-mixing process through flow transverse to downstream fluid streamlines in curved channels. In order to design the curved channels, Dean number should be considered in eq. S2 in addition to channel dimensions and flow rate:

$\text{Re=}\frac{\text{ρ}U_{f}\text{D}_{\text{h}}}{\text{μ}}$ (S1)

$\text{De=}\frac{\text{ρ}\text{U}_{\text{f}}\text{D}_{\text{h}}}{\text{μ}}\sqrt{\frac{\text{D}_{\text{h}}}{\text{2R}}}\text{=Re}\sqrt{\frac{\text{D}_{\text{h}}}{\text{2R}}}$ (S2)

where ρ is density of the aqueous solution, U_f_ is the velocity of the flow, D_h_ is the channel hydraulic diameter. The inertial lift forces dominate the particle behavior only when this particle Reynolds number is of order 1^2^ which could be achieved in laminar microfluidic devices.^3^

Figure 1S(a) showed the weight ratio of the photo-reduced Pt particles on the TNS with different feeding rate. With the increasing of the feeding rate, the weight ratio of the Pt was declined account for poor irradiation time. Lower feeding rate result in the higher deposition Pt particles until completely reaction of PtCl_6_^2-^. However, the lower rate could lead to a blockage in the microchannel in our experiment, because the TNS particles were not absolutely monodisperse in the aqueous solution. Figure 1S(b-c) were the HRTEM of the Pt/TNS-S and Pt/TNS, respectively.

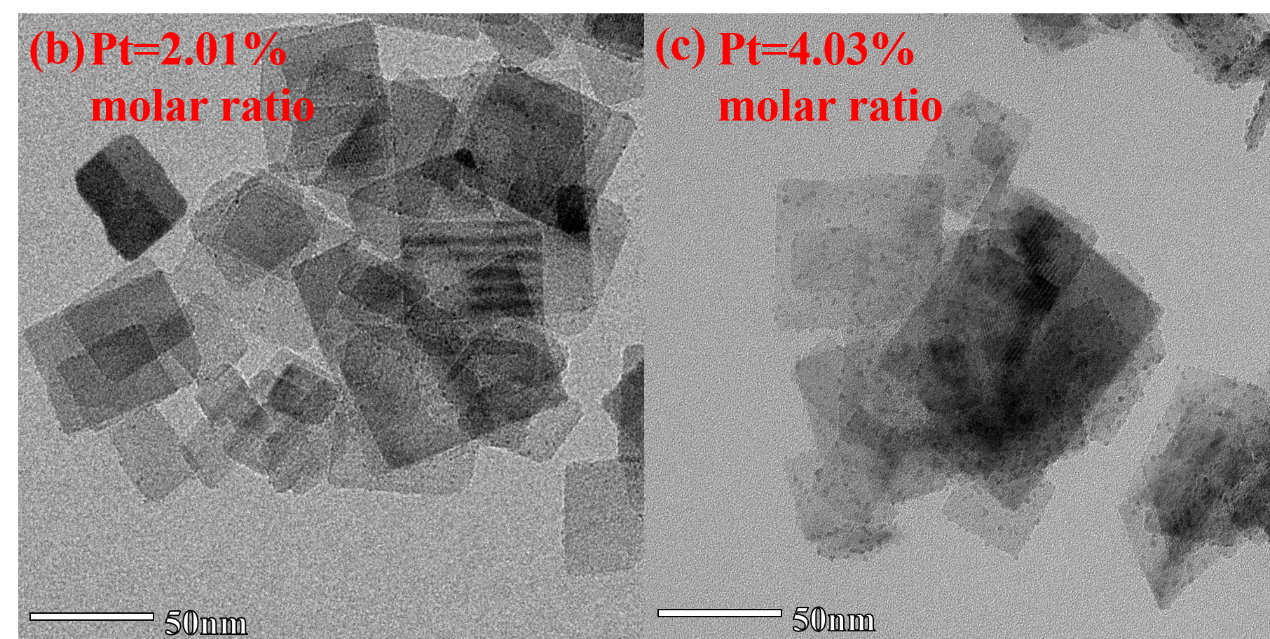


**Figure 1S. The diagram of (a) the molar ratio of the photo-reduced Pt particles on the TNS with variable feeding rate and (b-c) HRTEM of Pt/TNS-S and Pt/TNS, respectively.**

**S2. Characterization Data**

Raman spectrum illumination by 532nm was demonstrated in Fig. S1. to detect the structure of the sample. All samples were found to have similar characteristic peaks at 139, 392, 515 and 637cm^-1^ with Raman shift assigned to E_g(1)_, B_1g_, A_1g_+B_1g_ and E_g (2)_, indicating the presence of anatase TiO_2_, which were correlated with the diffraction peaks of anatase in the XRD patterns. Among those, the E_g_ vibration mode was mainly caused by bending vibration of the surface O-Ti-O bonds with fixed titanium atom, while the other three vibration modes were related to the Ti-O-Ti bonds with the fixed oxide atom.^4, 5^ In fact, Pt and surface silane coupling reagent were not clearly identified since the weak Raman scattering of those materials did not cause significant shift of the spectrum curves.

**Figure S2.** **Raman spectrum of TNS, Pt/TNS and J – Pt/TNS stimulated by 532nm laser.**

The amount of grafted TEFS on J-Pt/TNS could be determined by thermal gravimetric analysis. As shown in Figure S2, the temperature range of catalyst weight loss was observed from 100^o^C to 600^o^C at a heating rate of 10^o^C/min in N_2_. Under continuous oxidative heating, the weight loss of Pt/TNS was relatively stable with the changing temperature (within ca.3%). Such slight decline was owing to the limited absorbed water on the surface and the thermal decomposition of the residual reagents during the sample preparation. When the temperature was below 294^o^C, the weight loss of J-Pt/TNS was lower than that of Pt/TNS, which was probably due to the reduced absorbed water caused by surface hydrophobic modification. When the temperature reached 400^o^C, the weight loss of J-Pt/TNS became fast. After continuing to increase by about 50^o^C, the thermal weight loss reached ca. 2.5% showing the rapidly thermal decomposition of the surface grafted silane coupling agent ^6^.

**Figure S3. TGA and DTG curves of Pt/TNS and J-Pt/TNS.**

**Figure S4. Mean diameter of Pt particles of Pt/TNS**

References

(1) Thiele, M.; Knauer, A.; Malsch, D.; Csáki, A.; Henkel, T.; Köhler, J.M.; Fritzsche, W. Combination of microfluidic high-throughput production and parameter screening for efficient shaping of gold nanocubes using Dean-flow mixing. *Lab Chip*. 2017, *17*(8), 1487-1495.

(2) Leal, L.G. Particle Motions in a Viscous Fluid. *Annu. Rev. Fluid Mech.* 1980, *12*(1), 435-476.

(3) Wang, C.; Sun, S.; Chen, Y.; Cheng, Z.; Li, Y.; Jia, L.; Lin, P.; Yang, Z.; Shu, R. Inertial particle focusing and spacing control in microfluidic devices. *Microfluid. Nanofluid.* 2018, *22*(3).

(4) Jiao, J.; Wei, Y.; Zhao, Y.; Zhao, Z.; Duan, A.; Liu, J.; Pang, Y.; Li, J.; Jiang, G.; Wang, Y. AuPd/3DOM-TiO_2_ catalysts for photocatalytic reduction of CO_2_: High efficient separation of photogenerated charge carriers. *Appl. Catal., B* 2017, *209*, 228-239.

(5) Tahir, M.; Tahir, B.; Amin, N.A.S. Synergistic effect in plasmonic Au/Ag alloy NPs co-coated TiO_2_ NWs toward visible-light enhanced CO2 photoreduction to fuels. *Appl. Catal., B* 2017, *204*, 548-560.

(6) Yang, W.; Zhang, Y.; Yuen, A.C.; Chen, T.B.; Chan, M.; Peng, L.; Yang, W.; Zhu, S.; Yang, B.; Hu, K.; Yeoh, G.; Lu, H. Synthesis of phosphorus-containing silane coupling agent for surface modification of glass fibers: Effective reinforcement and flame retardancy in poly(1,4-butylene terephthalate). *Chem. Eng. J.* 2017, *321*, 257-267.
